# Aqueous synthesis of a small-molecule lanthanide chelator amenable to copper-free click chemistry

**DOI:** 10.1371/journal.pone.0209726

**Published:** 2019-03-27

**Authors:** Stephanie C. Bishop, Robert Winefield, Asokan Anbanandam, Jed N. Lampe

**Affiliations:** 1 Department of Pharmacology, Toxicology and Therapeutics, The University of Kansas Medical Center, Kansas City, KS, United States of America; 2 High Field NMR Core Facility, Center for Drug Discovery and Innovation, University of South Florida, Tampa, FL, United States of America; Aligarh Muslim University, INDIA

## Abstract

The lanthanides (Ln^3+^), or rare earth elements, have proven to be useful tools for biomolecular NMR, X-ray crystallographic, and fluorescence analyses due to their unique 4*f* orbitals. However, their utility in biological applications has been limited because site-specific incorporation of a chelating element is required to ensure efficient binding of the free Ln^3+^ ion. Additionally, current Ln^3+^ chelator syntheses complicate efforts to directly incorporate Ln^3+^ chelators into proteins as the multi-step processes and a reliance on organic solvents promote protein denaturation and aggregation which are generally incompatible with direct incorporation into the protein of interest. To overcome these limitations, herein we describe a two-step aqueous synthesis of a small molecule lanthanide chelating agent amenable to site-specific incorporation into a protein using copper-free click chemistry with unnatural amino acids. The bioconjugate combines a diethylenetriaminepentaacetic acid (DTPA) chelating moiety with a clickable dibenzylcyclooctyne-amine (DBCO-amine) to facilitate the reaction with an azide containing unnatural amino acid. Incorporating the DBCO-amine avoids the use of the cytotoxic Cu^2+^ ion as a catalyst. The clickable lanthanide chelator (CLC) reagent reacted readily with *p*-azidophenylalanine (paF) without the need of a copper catalyst, thereby demonstrating proof-of-concept. Implementation of the orthogonal click chemistry reaction has the added advantage that the chelator can be used directly in a protein labeling reaction, without the need of extensive purification. Given the inherent advantages of Cu^2+^-free click chemistry, aqueous synthesis, and facile labeling, we believe that the CLC will find abundant use in both structural and biophysical studies of proteins and their complexes.

## Introduction

Lanthanides, or rare-earth metals, are useful tools for fluorescence, X-ray crystallography, NMR, and mass cytometric analyses of proteins and their complexes [1–9]. Lanthanides have broad Stokes shifts, long fluorescence lifetimes, narrow emission peaks, resistance to photobleaching, and excitation/emission maxima disparate from the endogenous fluorophores tryptophan, phenylalanine, and tyrosine [10–14]. In addition, lanthanides’ unique paramagnetic properties may be utilized in protein NMR applications that expand the utility of NMR to larger proteins or protein complexes beyond that of conventional approaches [15–17]. Because of their ability to induce long-range paramagnetic shifts, incorporation of lanthanides facilitates examination of proteins of nearly 60 kD, twice the size of those garnered using conventional NMR approaches [15–17]. Finally, lanthanides’ unique spectroscopic properties lend to their use in time-resolved fluorescence and anisotropy analyses [18], augmenting the repertoire of tools for drug candidate screening against complex receptor targets [19]. Collectively, although these methods can be used to evaluate protein dynamics and protein-peptide interactions, site-specific incorporation is needed to fully harness the lanthanides’ unique paramagnetic and spectroscopic properties for structure determination and analysis [15–17]. Furthermore, the requirement of a chelating moiety to rigidly orient the lanthanide have reduced their usefulness in these applications.

Numerous strategies have been adopted to incorporate lanthanide chelators into target proteins, including genetic insertion of a lanthanide binding tag (LBT) [2]. However, the LBT’s rather large size, which may interfere with proper folding and/or function of the target protein, has prompted efforts to pursue small molecule lanthanide chelators [20–23]. Small molecule lanthanide chelators such as ethylenediaminetetraacetic acid (EDTA) and its derivatives 1,4,7,10-tetraazacyclododecane-1,4,7,10-tetraacetic acid (DOTA) and diethylenetriaminepentaacetic acid (DTPA) exhibit attomolar affinity for lanthanide metals and have been used for decades for their superior luminescence intensities [11, 24], and more recently, for NMR experiments [22].

To incorporate lanthanides into target proteins, these small molecule chelators and their analogs have been modified with nucleophilic alkylating agents to react with the free thiol group of a protein’s available cysteines [20–23, 25]. Where only one reactive cysteine is present, lanthanide paramagnetism can generate peak shifts of substantial magnitude and facilitate deconvolution of NMR spectra to yield structural information [4]. In many cases, however, the target protein may contain multiple cysteines, and/or they may form disulfide bonds or other stabilizing secondary structures, thereby precluding insertion or substitution of cysteine for site-specific incorporation of a lanthanide chelator. These considerations have limited the useful range of these particular reagents.

More recently, there has been increasing interest in utilizing click chemistry for incorporation of small molecule lanthanide chelators into proteins and other biomolecules [18, 26–31]. However, the syntheses of many of these “clickable” lanthanide chelators typically occurs in organic solvent [26, 29, 31–33], requires numerous steps [28–31, 33], and demands a copper catalyst for the click reaction [29, 34, 35], all of which have disadvantages when attempting conjugation to biologically active molecules. The requirement of a copper catalyst is particularly problematic for *in vivo* labeling experiments, given copper’s known cytotoxicity [36]. Furthermore, copper exhibits a high affinity for lanthanide chelators, making it difficult to remove from the chelator following the coupling reaction [31, 34].

Newer approaches exploit cyclooctyne-containing reagents which promote ring strain sufficient to initiate the [3 + 2] cycloaddition reaction between the azide and the alkyne [23, 31, 37–39]. As has been previously demonstrated by Agard and Bertozzi [37], the ring strain inherent in the cyclooctyne moiety spontaneously promotes the cycloaddition reaction with organic azides under aqueous reaction conditions without the need for a copper catalyst. This approach has distinct advantages for conjugations with biomolecules, particularly when conducting these reactions *in vivo*. Additionally, the essential ring strain of the cyclooctyne moiety can be stabilized by the addition of a pair of flanking phenyl rings, each on opposing sides of the cyclooctyne moiety. The resulting molecule, a dibenzylcyclooctyne, simultaneously offers sufficient shelf-life stability while catalyzing the click chemistry reaction in the absence of copper. Given that many varieties of dibenzylcyclooctyne reagents are now commercially available from a number of companies, including Click Chemistry Tools (https://clickchemistrytools.com/) and Millipore-Sigma (https://www.sigmaaldrich.com/), copper free click reactions can be performed to generate many different types of bioconjugates, potentially those including lanthanide chelating moieties [33], as demonstrated here.

To circumvent the issues described above, we reasoned that with the appropriate lanthanide chelation reagent, site-specific protein labeling could be achieved through site-directed mutagenesis and incorporation of the unnatural amino acid *para*-azidophenylalanine (paF) into the protein of interest [40]. Subsequently, copper-free click chemistry [41, 42] could be used to adduct a cyclooctyne-containing paramagnetic and fluorescent lanthanide chelator to the incorporated paF.

**Fig 1:**
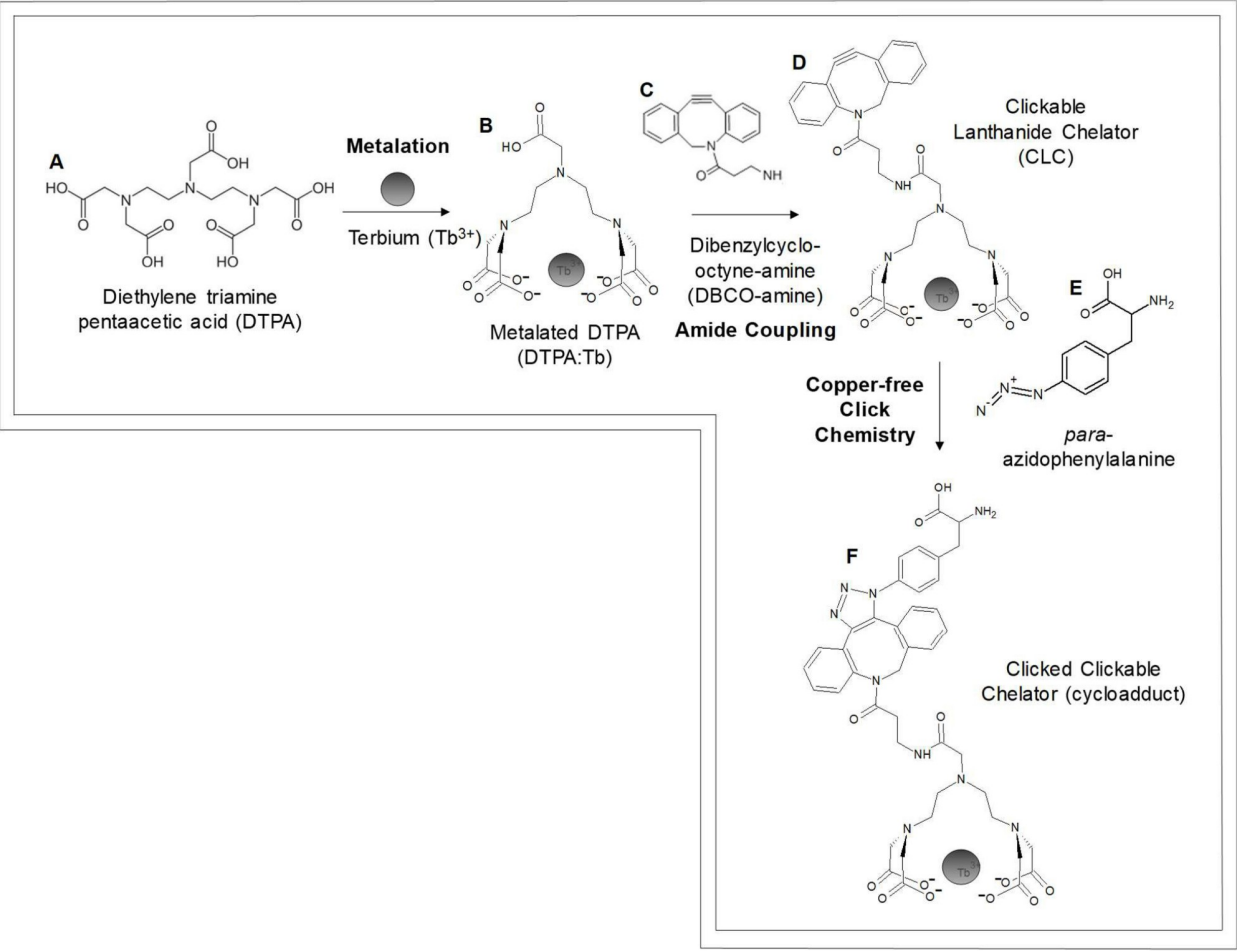
Generalized schematic of the aqueous synthesis of a small-molecule clickable lanthanide chelator (CLC, Molecule D). This is demonstrated utilizing copper-free click chemistry to yield the clicked clickable chelator (CCC, Molecule F), as described in this study.

Therefore, our goal was to synthesize, under aqueous conditions and using a limited number of steps, a cyclooctyne-containing small-molecule lanthanide chelator that would be amenable to copper-free click chemistry-mediated incorporation into a paF-containing protein. Our inquires resulted in a two-step aqueous synthesis of a lanthanide chelator (MW = 806.15 Daltons (Da)) for site-specific protein incorporation using dibenzylcyclooctyne-amine (DBCO-amine) and the small molecule lanthanide chelator DTPA (Fig 1).

## Materials and methods

### Chemicals and reagents

The dibenzylcyclooctyne-amine (DBCO-amine) used in this study was purchased from Click Chemistry Tools, (Scottsdale, AZ, USA). *Para*-azidophenylalanine (paF) was purchased from Bachem (Torrance, CA, USA). Terbium, 1,4,7,10-Tetraazacyclododecane-1,4,7,10-tetraacetic acid (DTPA), and all other reagents and bulk chemicals were purchased from Millipore-Sigma (St. Louis, MO, USA), unless otherwise noted. All solvents and chromatography media were of reagent, analytical, or HPLC grade.

### Nuclear magnetic resonance (NMR)

All ^1^H experiments were carried out at 298K using a Bruker (Billerica, MA, USA) Avance 600 MHz NMR spectrometer equipped with a TXI‐RT probe, using the PROHOMODEC pulse sequences for ^1^H detection. Data was processed using the Topspin v. 3.0 software package. Where necessary, additional processing was accomplished using the MestReNova (Escondido, CA, USA) software suite. Ten percent (v/v) D_2_O was used as a lock solvent for all compounds examined. All reported chemical shifts (δ) were measured in parts per million, as referenced to an internal standard of residual solvent.

### Analytical high performance liquid chromatography (HPLC)

A Shimadzu HPLC with Shimadzu SPD-M20A diode array detector, fluorescence detector, autosampler, and CLASS VP software (LabSolutions Lite, v. 5.52, Shimadzu, Kansas City, MO) were used for all HPLC analyses. For analytical HPLC, a Supelco 15 cm x 4.6 mm x 5 μm Supelcosil LC-18 column, Millipore-Sigma (St. Louis, MO) was used. Mobile phases to separate the copper-free click chemistry reaction product from DBCO-amine and paF consisted of A: 0.1% (v/v) trifluoroacetic acid (TFA) in water, B: 100% acetonitrile (ACN). The flow rate was maintained at 1.5 mL/minute; the gradient was 10% B from 0–1 minute, 10–90% B from 1–9 minutes, 90% B from 9–15 minutes, 90–0% B from 15–16 minutes, 0% B from 16 to 22 minutes. During each HPLC separation of the click chemistry reactions, 1 mL fractions were collected. Fluorescence was detected using a Shimadzu RF10AxL with data transfer to ML4.1 via Waters eSAT/IN A/D converter. Excitation wavelength was 350 nm and emission was monitored at 544 nm. To confirm the identity of the HPLC peaks that correspond to the cycloadducted product, fractions that exhibited absorbance at 280 and 310 nm (absorbance maxima for paF) and 454 (absorbance maxima for DBCO-amine in DMSO) were collected and dried under gentle nitrogen sparge then reconstituted in 40% TFA in water (0.1% (v/v)): 60% acetonitrile (100%) for small-molecule mass spectrometry, as described below. Mobile phases to separate copper-free click chemistry reactions between DBCO-DTPA:Tb and paF consisted of A: 0.1% (v/v) TFA in water, B: 100% ACN. The flow rate was 0.75 mL/minute; the gradient was 0% B from 0–1 minute, 0–100% B from 1–10 minutes, 100% B from 10–16 minutes, 100–0% B from 16–17 minutes, and 0% B from 17–22 minutes. One milliliter fractions that exhibited absorbance and retention times corresponding to the clickable chelator (DBCO-DTPA:Tb) and the clicked clickable chelator (the product of the click chemistry reaction between the clickable chelator (DBCO-DTPA:Tb) and paF) were collected through the elution phase.

### Anion exchange purification of metalated DTPA

Anion exchange chromatography was used to separate metalated DTPA from excess terbium trichloride used during metalation. The metalation incubation reaction (4 mL) was filtered using an Acrodisc 32 mm syringe filter containing a 0.2 μm Supor non-pyrogenic membrane. An ÄKTA Purifier 100 / 10 FPLC was used in conjunction with a HiTrap Q FF 5 mL GE Healthcare (Chicago, IL) anion exchange column, equilibrated with at least five column volumes (25 mL) of 100 mM MES (pH 5.5), prior to addition of the filtered metalation reaction. After application of the metalation reaction (4 mL), the SuperLoop injector column was protected from light with aluminum foil. Three column volumes (15 mL) of 100 mM MES (pH 5.5), were applied to the HiTrap Q FF anion exchange column, during which time 3.5-mL fractions were collected. Elution of metalated DTPA was achieved using 0–100% elution buffer, which consisted of 100 mM MES (pH 5.5), containing 1 M sodium chloride; the gradient was carried out over 8 column volumes during which time 3.5 mL fractions were collected. The Frac-920 carousel fraction collector and fraction collection tubes were protected from light with aluminum foil for the duration of the purification.

Purified samples and metalation reaction mixtures were further analyzed using a Fluoromax Fluoro-Hub from HORIBA Jobin Yvon (Edison, NJ), controlled with Origin FluorEssence software package (HORIBA Jobin Yvon), with an excitation wavelength of 350 nm and monitoring emission from 400–800 nm.

### UPLC-Mass spectrometry

A Waters (Milford, MA) SYNAPT HD hybrid quadrupole time of flight mass spectrometer coupled to a Waters Acquity Classic ultra-performance liquid chromatograph (UPLC) were used for LC-MS measurements. Instrument control, acquisition, and analysis were performed using Waters MassLynx software (version 4.1). The mass spectrometer was operated in sensitivity mode and the time to mass calibration was made with NaI cluster ions acquired under experimental conditions. Data was lock mass corrected against the doubly charged molecular ion of leucine enkephalin (YGGFL), intermittently acquired every 30 seconds using an auxiliary sprayer within the mass spectrometer’s source assembly. Mass spectra generated are the average of all mass spectral scans across an LC peak (acquired mass range: 200–1100 m/z; 1 second scans). The mass spectrometer conditions were as follows: electrospray ionization (ESI) negative ion mode, cone and desolvation gas (99.999% pure nitrogen) flows of 50 L/hr and 650 L/hr, respectively, desolvation and source temperatures of 350°C and source temperature of 120°C, respectively, capillary voltage of 2.5 kV, sample cone voltage of 30 V, and extraction cone voltage of 4 V for the exterior cone. For MS/MS experiments, the mass spectrometer was operated in data-independent mode with MS/MS acquisition mode initiated using a signal intensity trigger, a collision energy of 30 eV with ultrapure argon gas was admitted to the collision (trap) cell at 1.5 mL/min. Samples were resolved with a water/acetonitrile gradient through a Millipore Sigma (Burlington, MA) Supelco Supelcosil LC-18 column (15 cm x 4.6 mm x 5 μm) protected by a Phenomenex C18 Guard Cartridge. Mobile phase A was 0.1% (v/v) formic acid in water. Mobile phase B was acetonitrile with 0.1% (v/v) formic acid. A fifteen minute, 7-step gradient program was used: (1) 0–1 min isocratic hold at 5% B, (2) 1–2 min linear gradient to 25% B, (3) 2–8 min linear gradient to 40% B, (4) 8–10 min linear gradient to 99% B, (5) 10–12 min isocratic hold at 99% B, (6) 12–13 min linear gradient to 5% B, (7) 2 min isocratic hold at 5% B to equilibrate the column prior to next injection.

### Synthetic procedures

#### Metalation of diethylenetriamine-pentaacetic acid (DTPA)

**Fig 2:**
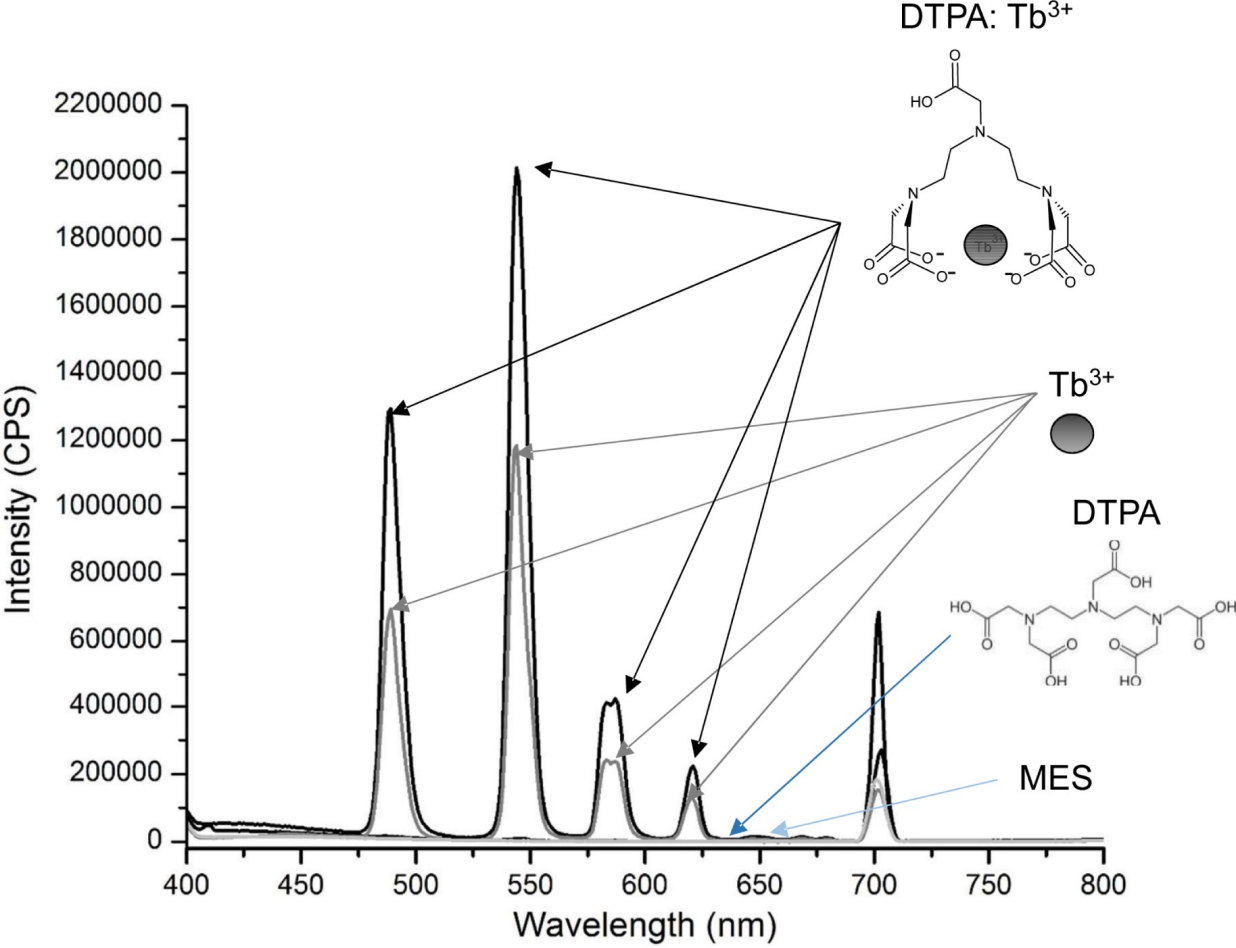
Steady-state fluorescence emissions spectra of metalated DTPA. Fluorescence emission spectrum observed upon metalation of DTPA by Tb^3+^. Fluorescence intensity, in counts per second (cps), of 100 mM MES, pH 5.5 (MES) (light blue arrow), 10 mM DTPA in 100 mM MES, pH 5.5 (DTPA) (dark blue arrow), 40 mM terbium trichloride in 100 mM MES, pH 5.5 (gray arrows), and 10 mM DTPA, 40 mM terbium trichloride in 100 mM MES, pH 5.5 (black arrows). The excitation wavelength was 350 nm; emission was monitored from 400–800 nm. The water Raman is evident at 702 nm. Peaks at 489, 544, 584, and 621 correspond to terbium fluorescence emission.

In our initial investigations, we observed that lanthanum, europium, and ytterbium all exhibited profound fluorescence intensity after incubation with DTPA, indicating that they were tightly complexed with the chelator. However, the paramagnetic lanthanide terbium was ultimately chosen due to its attomolar DTPA binding affinity [43], substantial fluorescence intensity [11, 14], ability to induce pseudocontact shifts [4, 7], and large paramagnetic relaxation enhancements (PREs) [4]. Initially, In order to prevent chelation of DTPA with other di- and trivalent cations, the first step of the synthesis involved metalation of DTPA with the lanthanide terbium (Tb^3+^) to generate DTPA:Tb (Fig 1B). Due to its attomolar affinity, terbium remained chelated throughout synthesis and purification, and is not likely to be displaced by di- and trivalent cations in subsequent click reactions. Metalation of DTPA with terbium was achieved under aqueous conditions using 2-(*N*-morpholino)ethanesulfonic acid (MES), a buffer devoid of free amines; MES was chosen to prevent off-target side reactions during the amide coupling step. Ten millimolar DTPA was dissolved in 100 mM MES, pH 5.5, with heat (75°C). DTPA was then metalated by incubating 10 mM TbCl_3_ and 10 mM DTPA at 75°C for 10 minutes in 100 mM MES, pH 5.5. ^1^H NMR analysis indicated near-quantitative complexation between Tb^3+^ and the DTPA chelator (Fig 2). Anion exchange chromatography coupled with fluorescence analysis was then used to purify the complex to >95% purity.

#### Synthesis of clickable lanthanide chelator (CLC) through amide coupling to DTPA:Tb

Metalation of DTPA with terbium exposed DTPA’s central carboxylic acid moiety for amide coupling with the DBCO-amine (Fig 1B) to generate the clickable lanthanide chelator (CLC, Fig 1D). Amide coupling was performed by combining DBCO-amine dissolved in DMSO (8.33% (v/v)) in 100 mM MES, pH 5.5, with 10 mM DTPA:Tb to achieve a final concentration of 50 mM DBCO-amine. 1-Ethyl-3-(3-dimethylaminopropyl)carbodiimide (EDC) was added to the amide coupling reaction until a final concentration of 50 mM was reached. The reaction was gently rocked, protected from light, at room temperature for 120 minutes. Upon completion, the entire reaction mixture was applied to a pre-equilibrated size-exclusion column containing G10 Sephadex resin (GE Healthcare, Chicago, IL), which excludes particles ≥ 700 Da, and therefore also the CLC (806.2 Da). Approximately 3.5-mL fractions were eluted from the G10 column using 100 mM MES, pH 5.5. Each fraction was evaluated using a Fluoromax 4 FluoroHub spectrofluorometer (HORIBA Jobin Yvon, Edison, NJ), with the Origin FluorEssence software package (HORIBA Jobin Yvon). Fractions were analyzed for fluorescence as follows: 1) excitation at 350 nm with emission monitored from 400–800 nm and 2) excitation at 259 nm with emission monitored from 300–800 nm. Fractions containing substantial fluorescence were lyophilized, reconstituted in mobile phase, and analyzed by UPLC-MS.

#### Copper-free Click Chemistry Reactions

Copper-free click chemistry was performed by combining 50 μM paF and 500 μM CLC (DBCO-DTPA:Tb) in 100 mM MES, pH 5.5. Reactions were gently rocked, protected from light, for 2 hours at room temperature. Subsequently, select aliquots were lyophilized then resuspended in 95:5 water:acetonitrile with 0.1% (v/v) formic acid; they were then analyzed by UPLC-MS as described above.

## Results

**Fig 3:**
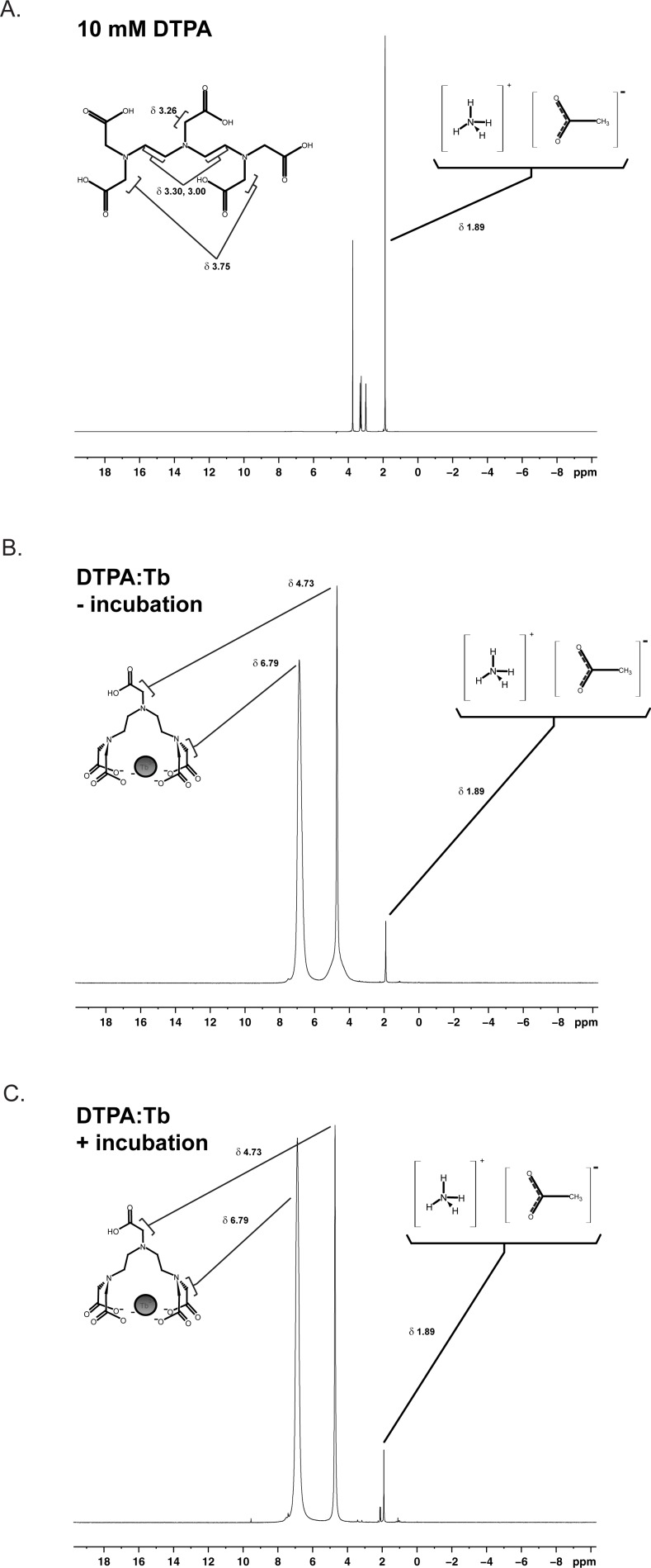
^1^H NMR spectra of DTPA chelation of Tb^3+^. ^1^H NMR spectra of 10 mM DTPA (top) and DTPA:Tb in 100 mM ammonium acetate, pH 5.5, before (middle) and after (bottom) incubation. ^1^H NMR analyses before and after incubation yielded nearly identical peak shifts. Compared to DTPA in ammonium acetate, DTPA:Tb yielded broadened DTPA peaks, indicating complex formation or metalation of terbium with DTPA.

The synthesis of the CLC began with a metalation reaction that involved metalating the commercially available lanthanide chelator DTPA with Tb^3+^. Metalation was evaluated using fluorescence (Fig 2) and ^1^H NMR (Fig 3). As demonstrated in Fig 2, neither MES buffer alone (light blue arrow) nor DTPA in MES (dark blue arrow) yielded substantial fluorescence emission from 400–800 nm, save for the water Raman peak (at 702 nm). DTPA with terbium trichloride in MES yielded sharp emission peaks at four wavelengths (489, 544, 584, and 621 nm; Fig 2; black arrows) that exhibited greater fluorescence intensity than did terbium trichloride alone in MES (Fig 2; light gray arrows). Furthermore, the ^1^H NMR spectra (Fig 3) revealed that, in the presence of terbium (Fig 3B and 3C), all DTPA ^1^H peaks are substantially line broadened and chemically shifted due to the paramagnetic nature of the terbium ion (e.g., δ = 3.75 ppm to δ = 6.79 ppm for the coordinated allylic amide protons and δ = 3.26 ppm to δ = 4.73 ppm for the uncoordinated carboxyamide protons) (Fig 3). The chemical shift and extensive line broadening is indicative of complex formation or metal coordination of terbium with the DTPA chelator.

Compared to samples that were not incubated, incubation of DTPA:Tb at 75°C for 8.25 hours did not increase fluorescence intensity for DTPA:Tb in 100 mM ammonium acetate, pH 5.5, or 100 mM MES, pH 5.5. NMR analyses before and after incubation in 100 mM ammonium acetate, pH 5.5, (Fig 3) yielded nearly identical spectra, suggesting that DTPA chelated the majority of the terbium ion (Tb^3+^) even without a lengthy incubation. Considering both the fluorescence and NMR results, the time-consuming incubation step at 75°C may not be essential to quantitative Tb^3+^ chelation by DTPA and may therefore be omitted or shortened. Subsequent metalations were therefore conducted using a 10-minute incubation at 75°C and a 1:1 molar stoichiometry of chelator:lanthanide. DTPA:Tb fluorescence emission at 489, 544, 584, and 621 nm was used to monitor progress through subsequent synthetic procedures.

**Fig 4:**
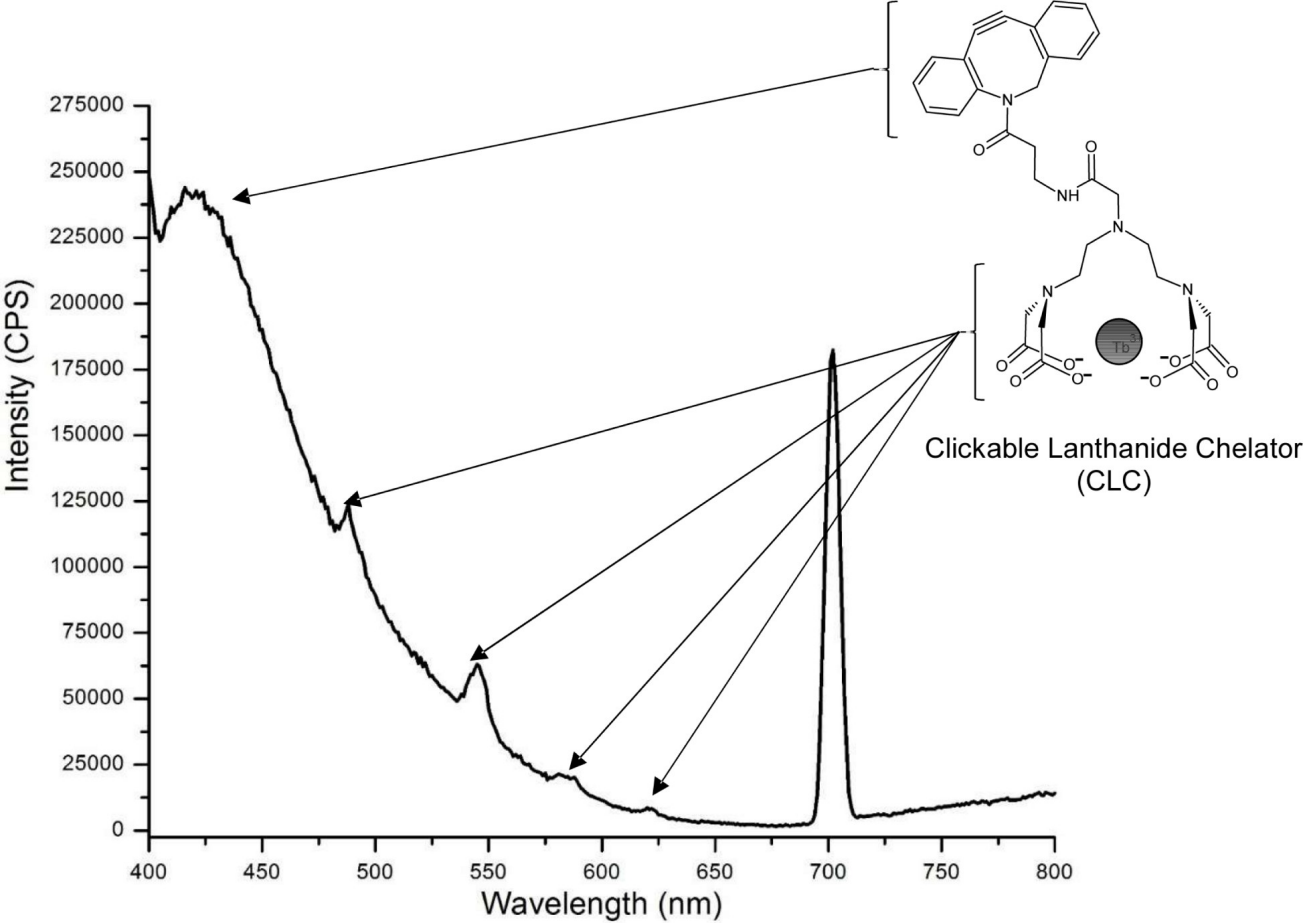
Fluorescence emission spectrum of metalated DTPA and clickable lanthanide chelator (CLC) after amide coupling and Sephadex gel column purification.

After completing amide coupling, the reaction mixture was applied to the pre-equilibrated G10 Sephadex column. As shown in Fig 4, Fraction 1, consisting of eluate from the void volume, exhibited substantial fluorescence at 429, 489, 544, 584, and 621 nm after excitation at 350 nm. The existence of this fluorescence signature in a fraction from the void volume suggested that it originated from a molecule with a molecular weight of ≥ 700 Da, as molecules larger than this are excluded from the resin pores. As a control, we examined DBCO-amine (193.5 μM) in 3.225% (v/v) DMSO in 100 mM MES, pH 5.5, which yielded an emission maximum at 423 nm with a broad shoulder with a maximum at 740 nm; the excitation maximum was 259 nm. Given that the emission profiles of DBCO-amine and DTPA:Tb are different from that of the CLC, this suggested that synthesis of a molecule that contains both DBCO-amine and DTPA:Tb could be evaluated after amide coupling by monitoring the unique fluorescence emission profiles of these moieties, which are only observable when both species were present. Based on the structure of the DBCO moiety and the fluorescence character of DBCO-amine alone, the fluorescence emission at 429 nm likely corresponds to the DBCO-moiety (Fig 4). This unique fluorescence signature (emission at 429, 489, 544, 584, and 621 nm) demonstrated that both DBCO- and metalated DTPA are present in one type of molecule that is larger than 700 Da (the pore size of Sephadex G10). No other fractions exhibited simultaneous emissions from both terbium and DBCO; as expected, however, several ensuing fractions exhibited fluorescence associated with only with the DBCO-amine, which corresponded to elution of excess DBCO-amine used during amide coupling. These data demonstrate that the first G10 Sephadex fraction possessed unique fluorescence properties that originate from CLC’s dual DBCO- and terbium constituents. To confirm the identity of the putative CLC product, UPLC-MS analyses were performed.

**Fig 5:**
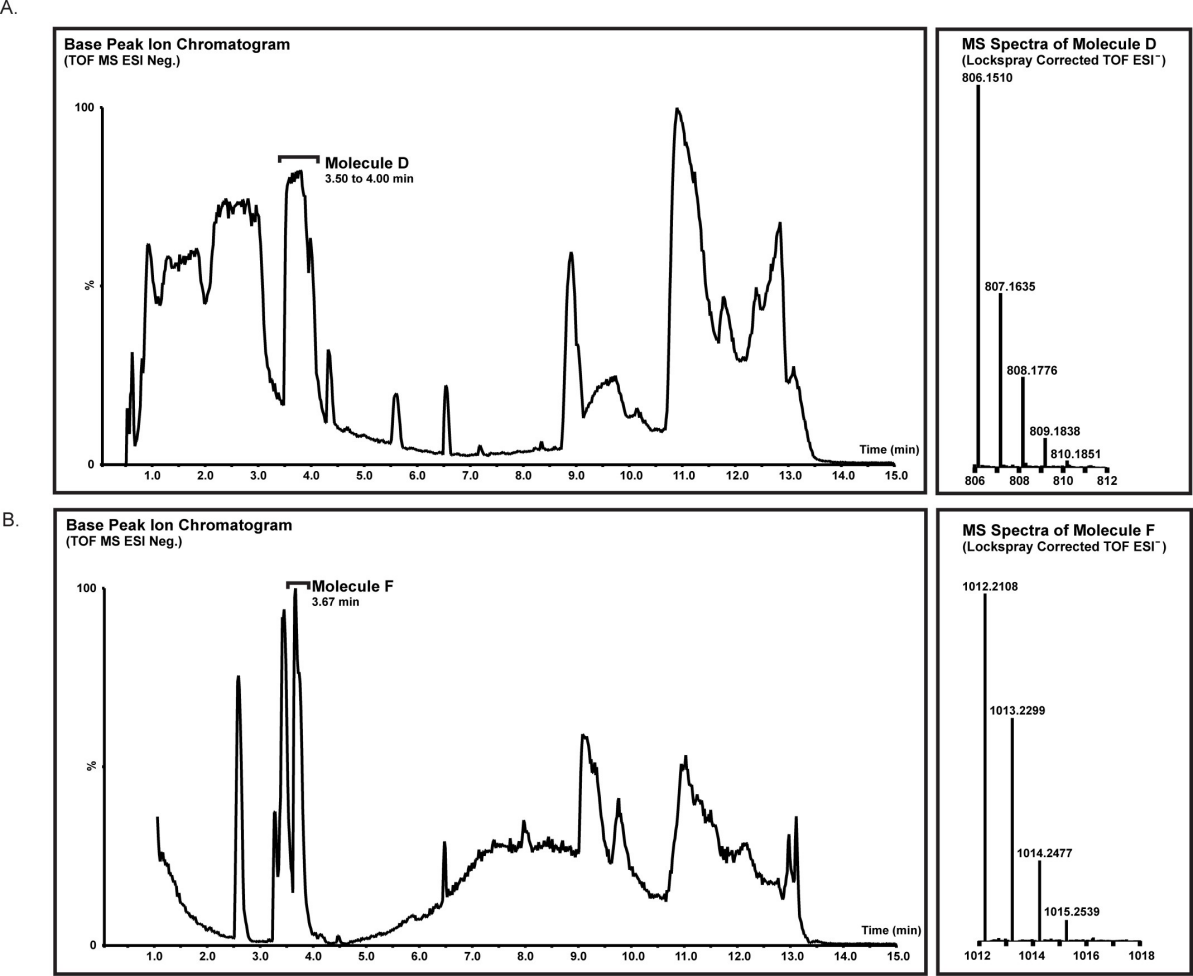
UPLC separation and MS spectra of clickable lanthanide chelator (CLC) and clicked clickable chelator (CCC) products.

**Fig 6:**
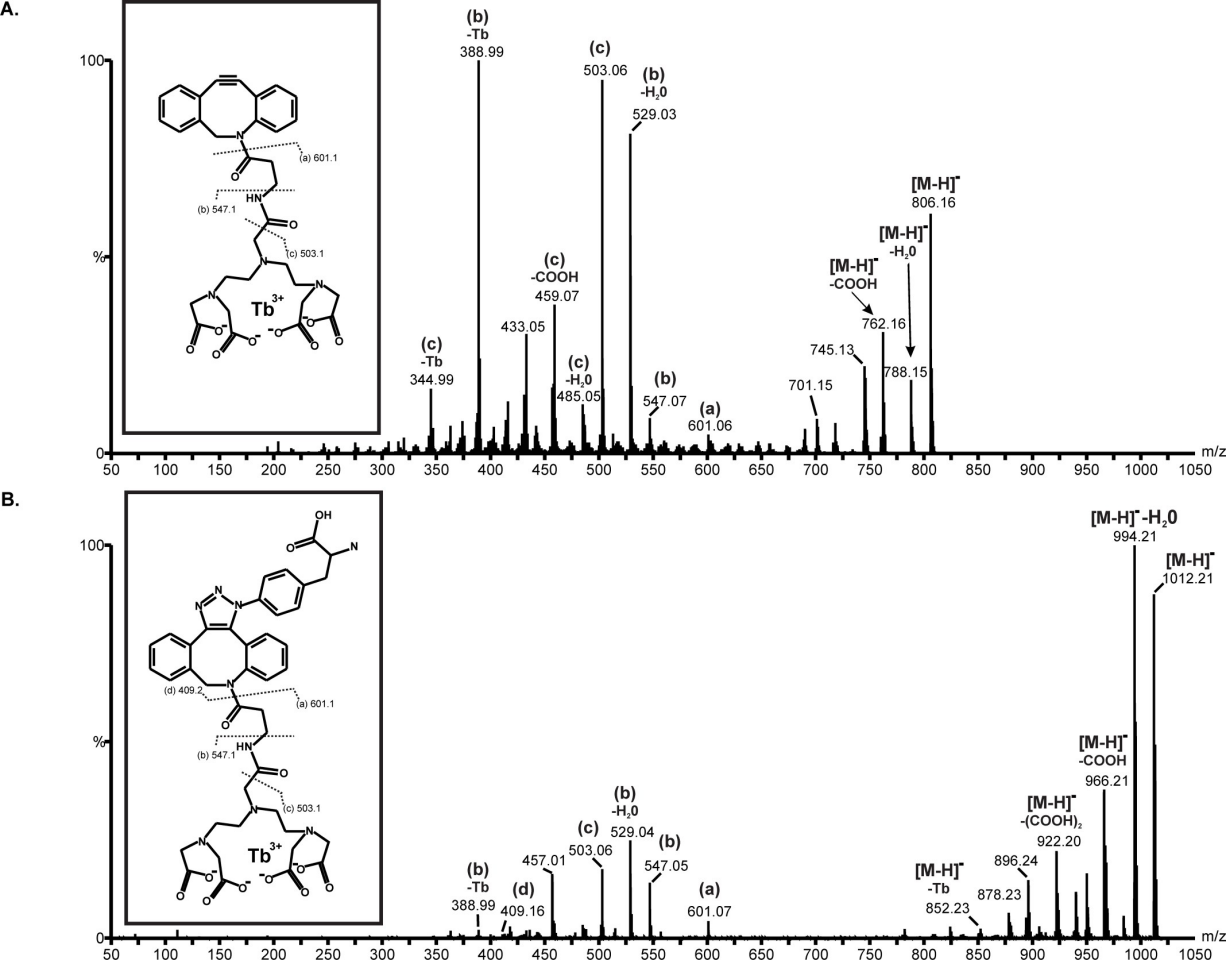
MS-MS analysis of clickable lanthanide chelator (CLC) and clicked clickable chelator (CCC) products.

UPLC separation of Fraction 1 yielded a peak at approximately 3.75-minutes retention time (Fig 5A; Molecule D) that exhibited fluorescence emission at 429 and 544 nm after excitation at 350 nm. As shown in Fig 5A (right panel) this peak was analyzed by mass spectrometry in negative ion mode and yielded a single peak at m/z = 806.2, the predicted theoretical isotopic mass of the CLC (Fig 1; Molecule D). (Note that negative ion mode was utilized due to significant signal suppression for the Tb^3+^ in positive ion mode). In order to further characterize the identity of this compound, MS-MS analysis was performed on the UPLC-MS product ion (Fig 6A). This resulted in the identification of three daughter ions with masses of m/z = 601.1 (a), 547.1 (b), and 503.1 (c), which corresponded to the Tb:DTPA-carboxyamide (a), Tb:DTPA-amide (b), and Tb:DTPA (c) moieties, respectively. These fragment ions were positively assigned to the putative structure of Molecule D, the CLC (Fig 6A inset).

To demonstrate the utility of adducting the CLC into a protein with a site-specifically-incorporated unnatural amino acid, a copper-free click chemistry reaction was performed between the CLC and paF; this generated the clicked clickable chelator (CCC), a cycloadducted chimeric product as shown in Fig 1, Molecule F. UPLC separation of an aliquot of a 10:1 molar stoichiometry CLC:paF copper-free click chemistry reaction yielded a peak with a retention time of 3.67 minutes that exhibited fluorescence emission at 544 nm after excitation at 350 nm. Negative ion mode UPLC-MS analysis of this peak yielded a single distinct product ion at the predicted theoretical isotopic mass for the clicked product (Molecule F), m/z = 1012.21, as depicted in Fig 5B (right panel). MS-MS fragmentation analysis of this product ion yielded three daughter ions, including two previously observed with the CLC (m/z = 547.1 (b), and 503.1 (c), respectively) and a novel ion of m/z = 409.2 (a), corresponding to the clicked DBCO-paF triazole moiety. The identification of these fragment ions allowed us to confirm the structure of the clicked CLC product, Molecule F (Fig 6B inset). The MS results corroborate the fluorescence data and demonstrate that the copper-free click chemistry reaction yielded the predicted cycloadduct (Molecule F). Unfortunately, given the limited amount of starting material, in the form of Molecule B, it was not possible to accurately estimate a final yield of the CCC. However, due to the ten-fold excess of CLC present, we estimate that the reaction proceeded to completion and utilized all available paF.

## Discussion

Lanthanide incorporation is a promising strategy to expand the utility of fluorescence and NMR in examining large biopolymers, offering the ability to evaluate protein dynamics [5], protein conformational changes [24, 44], and interactions of larger proteins than have previously been studied [7, 22]. However, the requirement for site-specific incorporation, coupled with the need for a lanthanide chelator, have limited its use. Currently, small molecule lanthanide chelators originate from multi-step syntheses in organic solvent that yield cysteine-reactive [20–23, 25] and other types of chelators that may [25] or may not [35] be site-specifically incorporated into the protein of interest.

While click chemistry has been employed to position lanthanide chelating bodies in proteins [18, 26–31], the difficultly of multi-step syntheses and the need for a copper catalyst have slowed the adoption of this approach for lanthanide incorporation into proteins. To overcome this, we have described a facile two-step synthetic method that relies on a lanthanide chelator (DTPA) that can be site-specifically incorporated into a protein of interest via an azide-containing unnatural amino acid (paF) employing the cyclooctyne moiety (as DBCO), without the need of a copper catalyst to enable the reaction. This small molecule chelator is amenable to copper-free click chemistry using relatively mild, aqueous reaction conditions and common commercially available reagents. This is of obvious advantage in that the reaction can be performed at room temperature, in physiological buffers, and free from copper, a known cytotoxicant [36]. Furthermore, one does not have to rely on the presence of an accommodating prosthetic group [30] or appropriately positioned cysteine residue [7] for incorporation of the lanthanide chelator to examine protein structure and/or dynamics. Another advantage to this approach is that, because of its relatively small size, this chelator is likely to elicit only minimal distortions on the target protein structure. This is of importance, because larger lanthanide binding elements, such as the LBT [44], have the potential to disturb secondary structure and therefore convolute the structural data obtained. Finally, the site-specific nature of incorporation afforded by the introduction of an unnatural amino acid into a specific location of the protein allows for distance constraints to be determined using pseudocontact shift parameters [33].

The usefulness of incorporating a lanthanide for protein structure determination has recently been realized with the elucidation of the structure of the cytochrome P450cam-putidaredoxin complex [7]. While the approach of Hiruma et al. relied on the specific incorporation of a lanthanide via adduction of a single cystine residue in the backbone of the P450cam enzyme, using paramagnetic NMR to yield 446 structural restraints, the authors were able to determine the structure of a region of the protein that was not originally observable in the crystal structure [7]. Additionally, their data also demonstrated the presence of a minor state or set of states of the complex in solution, which the authors attributed to the presence of an encounter complex between the two proteins. Future studies of this type would no doubt be aided by the precision combination of click-chemistry and site-specific incorporation afforded by the CLC that we have described. However, for this particular application to be successful, rigidity would need to be introduced into the chelation reagent (Molecule B) in order to elucidate the orientation of the lanthanide. We are exploring this possibility through the use of other chelation and conjugation reagents.

Another intriguing application of this technology is in the production of targeted molecular imaging agents [28, 29, 31, 45]. Since the DOTA chelation moiety efficiently chelates other lanthanides, it is likely that it will also chelate many of those used in PET and other types of radioimagining, such as ^111^In [31]. Additionally, the CLC compound may also be an effective MRI contrast agent if incorporated into appropriate *in vivo* imaging agent designs. Further demonstrating the utility of the CLC in this role is an area of active research in our laboratory.

We also expect that metalation with other lanthanides may be useful to capitalize on each lanthanide’s unique fluorescence and paramagnetic properties for use in protein structure and dynamics analyses. Future studies will include measuring the proton nuclear magnetic relaxation dispersion of the “clicked” complex with different lanthanides to confirm this capability.

While the initial synthetic efforts described herein have been small scale, using limited quantities of reagent, they afford a proof-of-principal demonstration of the feasibility of such an approach and lay the groundwork for efforts by our group and others to capitalize on improving the efficiency and scale of the synthesis as outlined. We are currently planning a larger scale synthesis of the CLC molecule for future studies, such as those mentioned above. Additionally, experiments involving adducting this chelator into azide-containing peptides and proteins are in progress, as we have already had success with incorporating the DCBO compound directly into proteins containing the paF unnatural amino acid.

Finally, because other carboxylic acid lanthanide chelators are commercially-available, alternative small molecule chelators may be synthesized using the strategies described here.

## Conclusion

We anticipate that these approaches may be suitable for use with azides and alkynes on a variety of different unnatural amino acids and fluorescent and paramagnetic labels, ideally generating many unique combinations of probes for evaluating proteins by fluorescence, X-ray crystallography, and NMR. In conclusion, we believe that the efforts described here yield a promising new tool to expand analysis of proteins with these biomolecular approaches.

## Abbreviations

CLC: clickable lanthanide chelator
CCC: clicked lanthanide chelator
DBCO: dibenzylcyclooctyne-amine
DOTA: 1,4,7,10-tetraazacyclododecane-1,4,7,10-tetraacetic acid
DTPA: diethylenetriaminepentaacetic acid
EDC: 1-ethyl-3-(3-dimethylamino-propyl)-carbodiimide
EDTA: ethylenediaminetetraacetic acid
MES: 2-(*N*-morpholino)ethanesulfonic acid
paF: *para*-azidophenylalanine
